# Understanding Autism as a Condition in Mental Health Clinical Practice: Clinical Perspectives from a Youth Early Psychosis Service

**DOI:** 10.1007/s10597-024-01433-w

**Published:** 2025-01-08

**Authors:** Caillin Porter, Richard Whitehead, Liza Hopkins

**Affiliations:** 1 Alfred Health Headspace, Melbourne, Australia; 2 Deakin University, Melbourne, Australia; 3 Alfred Health Child and Youth Mental Health Service, Melbourne, Australia

**Keywords:** Autism, Autism traits, Early psychosis, Mental health services, Young people

## Abstract

Autism is a rapidly growing phenomenon, with rates of diagnosed autism in the community rising every decade. Autism and traits of autism are also regularly part of presentation at youth mental health services, including early psychosis services. In early psychosis services young people’s symptoms tend to be formulated through a psychosis lens, rather than a neurodevelopmental lens which can lead to unnecessary medicalised treatment, and treatment plans that do not consider the possible impact of neurodiversity. The following paper explores autism and traits of autism in relation to youth early psychosis, examining the complexity in accurate formulation, and the possible impacts for young people. Future directions for how services can address this issue and more effectively tailor treatment to young people are also discussed.

Autism is a rapidly growing phenomenon with rates of diagnosed autism in the community increasing every decade (Centre of Disease Control & Prevention, 2024). Autism and traits of autism are also regularly part of presentation at mental health services, including youth early psychosis programs (Sunwoo et al., 2020). Client presentations to early psychosis services vary considerably, however hearing voices and unusual sensory experiences characterise most referrals. When viewed cross-sectionally, these experiences are often formulated through a psychosis lens, however, if similar experiences are identified over the course of a person’s life, understanding these experiences through a neurodevelopmental lens such as autism may be more helpful. Due to a potential lack of confidence in identifying and treating autism among clinicians working with adolescents and adults, signs of autism may often be missed or not considered in case formulation and treatment planning. The aim of the following paper is to examine the rapidly evolving understanding of autism in young people and what this means for youth mental health services. We then examine the overlap between autism traits and early experiences of psychosis and how the understanding and misunderstanding of these two conditions may affect the support that young people receive in early psychosis services. Finally, we propose the need for consideration of autism and traits of autism when providing effective case formulation and treatment planning to improve outcomes for young people presenting to early psychosis services.

## Autism

Autism is currently defined as a congenital neurodevelopmental condition which has a significant impact on development in early childhood and throughout life (APA, 2013). Autism can present in many ways, but is often characterised by features such as social and communication differences (Lee et al., 2020; Yirmiya et al., 2006) and hyper- or hypo-sensitive sensory processing (Ben-Sasson et al., 2009; Sinclair et al., 2017), with some autistic people having a heightened pre-occupation on topics/interests (Van Steensel et al., 2011) and preferences for sameness/difficulties with change (Gotham et al., 2013).

In psychiatry, the definition of autism has been repeatedly revised over the past 40 years (Volkmar, 2016; Volkmar & McPartland, 2014), and the incidence rates are trending from the condition being seen as rare in the 1990s, to relatively common at present. The incidence rates of autism over the last three decades have grown from 1 in 2500 in the 1990s, to 1 in 150 in 2000, 1 in 100 in 2010, 1 in 60 in 2014, 1 in 44 in 2021, and 1 in 36 in 2023 (Centre of Disease Control & Prevention, 2024). Even with this rapid increase in diagnosis, there remains a large cohort of adults and young people, known as the ‘lost generation’, who have autism traits but have not received a diagnosis (Lai & Baron-Cohen, 2015).

The presentation of autism can be difficult to understand without targeted training and experience, as autism can be comprised of a combination of the above diagnostic indicators, as well as many indicators not considered diagnostic, leading to the sweeping differences seen between any two autistic individuals, and the concept of ‘spectrum’ (Mottron & Bzdok, 2020). Due to the heterogeneous presentations of autistic individuals, and systemic barriers involved with receiving an autism assessment, diagnosing autism can be challenging (Martinez et al., 2018). Typically, a high standard assessment involves independent input of around three separate specialists (e.g., a combination of paediatrician, psychiatrist, clinical psychologist, occupational therapist and speech therapist). While multidisciplinary standards of autism assessment can lead to high quality assessments, the accessibility of such assessments can be low (Howes et al., 2021). In many instances (for example remote communities, migrant communities, and low-income families), accessing autism specialists is very challenging, thus many young people are missing opportunities for assessment.

Due to this specialised nature of autism assessment, there are a range of identified barriers to some people receiving autism diagnoses (Martinez et al., 2018). Barriers such a long wait times, problems finding providers (Martinez et al., 2018), and an identified lack of clinician knowledge in first identifying the signs of autism (Howes et al., 2021) can all contribute to people not receiving the appropriate support. For many adults seeking an autism assessment, other prohibitive factors such as cost, and fear of not being believed can also stand as barriers to receiving support (Huang et al., 2022).

Beyond these documented barriers in accessing autism assessments, there may also be many people who display traits of autism but who do not meet the criteria for an autism spectrum disorder (ASD) diagnosis (Defresne & Mottron, 2022). The current diagnostic criteria, while describing ASD as a lifelong condition, requires there to be an “impairment” to receive a diagnosis of ASD. The current criteria, while being helpful, can be limited by the impairment-scope in helping clinicians (and the community) to understand and appreciate the fundamental qualities of autistic people (Jellett & Muggleton, 2022). The impairment or disorder lens can fail to take into account a more nuanced understanding of autism and autism traits, and can mean that many people who display traits of autism, but who have not received a diagnosis, may not have this factored into their formulation and the treatment they receive.

A recent movement, largely driven by people with autism and their families, seeks to redefine autism away from a *disorder* and toward a *condition* (Hus & Segal, 2021) which may or may not include impairment. This is supported by research showing a high occurrence of subclinical autism traits in family members of autistic individuals (Defresne & Mottron, 2022). The autism ‘phenotype’ is a term used to capture people who share these *subclinical* differences in social functioning, communication, and restricted interests and behaviours, usually with an implication of shared genetic markers with autism (Defresne & Mottron, 2022; Landry & Chouinard, 2016). As the difficulties observed in this group can be significantly milder, they do not fit within the ‘clinical cut-off’ of ASD as currently defined and these traits may not be part of assessments and treatment planning.

## Understanding Autism in Youth Mental Health Services

Another potential barrier to how autism and traits of autism are identified and treated in health services can be the differences in the way presentations are understood and discussed. For example, in some areas, child and adult psychiatry formulate and use diagnostic language differently (Winters et al., 2007). For example, behaviour and anxiety concerns in children may attract labels such as oppositional defiance disorder (ODD) and attachment disorders, however, if these problems persist into adulthood, the language and lens of adult psychiatry may shift toward personality disorders (PDs) such as borderline PD and antisocial PD. This difference in language and formulation can have implications in youth mental health care, where clinicians working in the adolescent/young adult age range may be influenced by their training in adult psychiatry. Formulation of mental health issues of young people through an adult psychiatry lens may then impact the treatment and support young people receive.

Training in identification of autism is considered a central requisite for most *childhood* mental health clinicians. However, this contrasts significantly with the knowledge base of *youth and adult* mental health clinicians, for whom experience, expertise, and confidence in this area can be low (Corden et al., 2022; Gallant et al., 2023; Maddox et al., 2020). As youth and adult mental health clinicians, they may be less likely to feel confident identifying autism, and less likely to formulate a client presentation through a neurodiverse lens.

In youth and adult mental health settings, it can be rare for clinicians to have expertise in developmental conditions such as autism (Lipinski et al., 2022). Even if learnt during professional training, the diagnostic criteria for ASD are focused on traits that are observable in young children, and in many cases, by adolescence the young person may have learned to mask their most obvious traits of autism (Belcher et al., 2022). Many clinicians struggle differentiating symptoms of autism from other mental health conditions (Au-Yeung et al., 2019) and may fail to consider autism traits when tailoring treatment plans for the client (Rosen et al., 2018).

Although clinicians may not identify indicators of an underlying autism presentation, this does not mean they are unsupportive, and they may still provide strong interpersonal support with positive outcomes for the client. However, there may be significant risks associated with clinicians missing an underlying autism presentation, particularly if this leads to unnecessary medicalised treatment of a misdiagnosed alternate mental illness such as schizophrenia or bipolar disorder (Au-Yeung et al., 2019; Luciano et al., 2014; Van Schalkwyk et al., 2015).

This may be especially true for young people presenting to an early psychosis program who, if viewed through a childhood developmental lens, may be more likely attract formulations (amongst others) associated with neurodevelopmental conditions, while from an adult lens are perhaps more likely to attract formulations such as ‘developing traits of BPD’, ‘early psychosis’, and ‘at risk of bipolar disorder’. Each lens may be potentially beneficial for the client, but the course of treatment can be vastly different.

## Autism and Mental Health

It is well established that adolescents with an underlying autism presentation have clusters of challenges which can overlap many acute mental health conditions (Chisholm et al., 2019). For example, poor emotion and physical regulation strategies (e.g. self-harm) and interpersonal difficulties, common experiences of an adolescent with underlying autism, may be formulated through the lens of a developing borderline personality disorder (BPD; Iversen & Kildahl, 2022). Restrictive or excessive eating in times of stress could be formulated as an eating disorder, but could also be driven by underlying autism (Brede et al., 2020). Similarly, clients presenting with hearing voices may be treated or assessed as having psychosis or at risk of developing psychosis (Au-Yeung et al., 2019; Chisholm et al., 2019), despite autistic people being significantly more likely to experience sensory anomalies such as hearing voices (APA, 2013).

In addition to the overlap between symptoms of autism and mental health diagnosis, people with autism are significantly more likely to experience mental health issues such as mood disorders, behavioural and emotional disorders, and schizophrenia than the general population (Rim et al., 2023). It has also been demonstrated that young people with traits of autism, even if falling below clinical cut-off points, may still be at a significantly higher risk of developing mental health difficulties, such as depression and suicidality, and be over-represented in mental health services (Chisholm et al., 2019; Dell’Osso et al., 2019).

For some, the traits of autism are obvious and functionally inhibiting during infancy and early childhood, with these people tending to gain attention from health services and educational providers, as parents are alert to signs of difference, leading to assessments and possible diagnosis. The emphasis on early detection of autism has had many benefits (Bryson et al., 2003; James & Smith, 2020), such as an increase in identification of autism and associated targeted emotional, developmental, relational, functional, and educational supports for young children (Elder et al., 2017). However, for other young people, the markers of autism in their developmental trajectory may not be as obvious or as easy to identify (Jellett & Muggleton, 2022; Landa et al., 2013).

For young people who may not be initially identified as autistic, adolescence can present additional challenges. Entering adolescence, the education system becomes less flexible and nurturing, and the social dynamics and developmental expectations become exponentially more abstract and complicated. For children that have previously been coping, navigating the social and biological tasks associated with adolescence can precipitate clinical concerns which may be the first clinical markers of an underlying autism condition (Aggarwal & Angus, 2015; Picci & Scherf, 2015; Van Schalkwyk et al., 2015).

Seeking support from a mental health setting is often precipitated by an increase in distress or change in functioning, and the age of onset for mental health challenges can often echo the age when individuals with autism and autism traits experience more challenging behaviours and experiences (Aggarwal & Angus, 2015). Mood fluctuations, self-harm, suicidal thinking or attempting, existential dilemmas, depression, bullying and social conflict, repetitive patterns of thinking and behaving, and a lack of behavioural flexibility can lead to a first presentation to a mental health setting, including early psychosis services.

## Autism and Psychosis

Historically, autism and psychosis have been intrinsically linked conditions. In the first half of the twentieth century, autism was understood as a *childhood expression of Schizophrenia* (Cappon, 1953; King & Lord, 2011). Stigma around autism was particularly amplified when the emotional detachment of some autistic children was erroneously formulated as resulting from cold parenting, with the term ‘refrigerator mother’ causing significant pain for many families (Jack, 2014). During the 1980s, psychiatry more clearly defined and delineated autism from psychotic illness (Volkmar & McPartland, 2014). Even though the understanding of autism as a separate nuanced condition has evolved, research, including genetic studies, continues to document the overlap between these two conditions (Colizzi et al., 2022; Guerra et al., 2024; Larson et al., 2017; Sunwoo et al., 2020; Zheng et al., 2018). Schizophrenia has also been considered as a neurodevelopmental disorder (De Berardis et al., 2021; Gupta & Kulhar, 2010), with genetic markers of schizophrenia being present before birth, and characteristics such as impaired social cognition being evident before the onset of first episode of psychosis (Healey et al., 2016). Statistics also support the overlap of these two conditions. People with autism are around 3.6 times more likely to be diagnosed with schizophrenia (Zheng et al., 2018). Retrospectively, of people with Schizophrenia, 30–50% also present with substantial symptoms of autism in early childhood (Kincaid et al., 2017). While the current diagnostic criteria of autism and psychosis have few similarities, in practice, the task of teasing apart these two conditions can be challenging (Ferrara et al., 2024; Ribolsi et al., 2022).

## Psychosis

Traditionally, psychotic symptoms tend to be easily defined. These can include unusual sensory experiences (typically auditory and visual, and clinically referred to as ‘hallucinations’) and unusual thought patterns, such as beliefs and preoccupations with unusual ideas, often with paranoid or grandiose themes (APA, 2013). The presence of these symptoms, usually in conjunction with an episode of crisis (acute stress/drug use) or change in functioning, typically culminates in the identification of a ‘psychotic episode’.

Psychotic symptoms, as defined above, are trans-diagnostic and experienced across the spectrum of clinical diagnoses, including people with autism (Volkmar & McPartland, 2014), trauma (Schäfer & Fisher, 2011; Spauwen et al., 2006), BPD (Schroeder et al., 2013; West et al., 2021), depression (Liu et al., 2023), and even in non-clinical groups (Park et al., 2022; Verdoux & van Os, 2002). As such, defining psychosis as an *illness* (i.e. Schizophrenia) is not straight forward (Seikkula, 2019; Verdoux & van Os, 2002). The occurrence of psychotic symptoms is associated with *increased risk* of developing a more enduring psychotic condition, however, confirmation bias can occur when a clinician/treating team relies on these symptoms alone to identify a client with a psychotic illness, rather than unpacking the diverse possible causes of these symptoms.

Complexities may also arise when the presenting symptoms may be indicative of more than one condition, including ASD (Ferrara et al., 2024). Due to the high comorbidity of autism traits and psychosis and the overlap in their presentation (Kincaid et al., 2017; Lee & Lee, 2023), clinicians may neglect symptoms of autism due to the diagnostic overshadowing of the symptoms aligning with a psychosis presentation (Au-Yeung et al., 2019). There is also a growing body of research suggesting the many shared neurobiological features of ASD and psychotic disorders, such as impaired social cognition (Barlati et al., 2020; Healey et al., 2016; Kincaid et al., 2017), which has also been observed in significant numbers in people presenting with early psychosis (see Healey et al., 2016 for a review), that can make the distinction between the two conditions even more difficult.

There is also evidence to suggest that some individuals originally diagnosed with a psychotic illness, may be better understood through a lens of autism (Au-Yeung et al., 2019; Dossetor, 2007; Van Schalkwyk et al., 2015). When re-analysing cases of young people diagnosed with psychosis, Dossetor (2007) found that clients’ presentation could be better understood in terms of their autism symptoms, such as repetitive behaviours and pre-occupations. Van Schalkwyk et al. (2015) also found evidence that individuals diagnosed with psychosis may have had better outcomes if their autism symptoms had been better understood and integrated into their treatment plans. A recent qualitative report by Au-Yeung et al. (2019) also found that people with autism felt they had been misdiagnosed with mental illness and that a lack of awareness about autism from mental health clinicians may have contributed to this experience. These findings do suggest that autism assessment needs to be part of all treatment plans involving psychosis, but suggests the importance of treatment teams to have dynamic discussions about client presentations, which capitalise on the depth of knowledge of both childhood *and* adult mental health clinicians and the potential for overlap between these two conditions.

## Observations from a Youth Early Psychosis Service

Youth early psychosis programs see young people who have experienced a first episode of psychosis (an acute episode of psychotic symptoms) or are considered at Ultra-High Risk (UHR) of developing a more enduring psychotic disorder. Some YP may present with an existing mental health diagnosis, including ASD, or this may be their first presentation to the mental health services. Psychotic symptoms are naturally the stand-out experience which lead to a referral to these programs. However, accompanying a referral to an early psychosis program is often the observation that the interpersonal and cognitive style of these clients often stands-out as different to that expected of similar aged peers. When viewed cross-sectionally, clients speaking about strange ideas, becoming distracted by their internal world or the sensory environment, and being withdrawn from, or inappropriate in, social relationships tend to attract hypotheses about psychosis. However, when similar patterns are identified over the course of a person’s lifetime, it may be helpful to also consider a neurodevelopmental lens.

One way of initiating clinical discussions which can capture neurodevelopmental conditions can be achieved through a clinician’s willingness to explore the concept of *difference* rather than *disorder* (Bottini et al., 2024). ‘Difference’ discussions may prompt information such as observations from family that their child’s development was characterised by atypicality, or anecdotes of unusual and quirky behaviours which may not naturally be discussed in a psychiatric setting. Using more neuro-affirming language such as ‘difference’ can provide an opportunity for a young person to voice their experiences of experiencing the social world differently to their peers without the stigma associated with more impairment-focussed language (Bottini et al., 2024). However, the focus of psychiatry can contribute to the tendency for clinicians to prompt clients and families to primarily explore topics of *distress and impairment.* Clients and families may not be encouraged to talk about *difference* and so they join the narrative of distress and impairment. Opportunities to explore potential underlying neurodiversity can be missed, which could potentially impact the quality of assessment and treatment plans.

Formulation of clients through an autism lens presents an alternate pathway to understanding clients’ experiences. For example, autistic people may have emotion and behaviour regulation difficulties which can mirror bipolar disorder or BPD, unusual sensory and cognitive experiences which can mirror a psychotic illness (Morimoto et al., 2021), and repetitive and restrictive cognitions and behaviours which can mirror OCD and eating disorders (Carpita et al., 2022). In these cases, thoughtful formulation and clinical discussion may contribute to a more nuanced understanding of the client that may help remove unhelpful psychiatric labels and potential medicalised treatments and promote psychosocial supports and treatments which are targeted to the underlying problems.

Interestingly, recent research suggests approximately 3.7% of young people with early psychosis are also diagnosed with autism (Sunwoo et al., 2020). However, it is difficult to determine the actual number of those that may not yet have been diagnosed, or may display sub-clinical traits of autism, as clinicians tasked with completing statistical data about their client’s clinical diagnosis may be less likely to identify a life-long neurodevelopmental condition which they have not assessed, and more likely to identify cross-sectionally assessed options such as anxiety, depression, psychosis, and bipolar disorder. Clinical documentation proformas rarely include spaces to encourage hypotheses which have not been assessed. An early psychosis program, by its very nature, primes staff members through training and data capturing to focus and intervene on psychotic symptoms. While well intended and beneficial for a significant proportion of clients, it may amplify a tendency to miss autistic features and only focus on the psychosis which, in some cases, could be detrimental.

Interestingly, research has also shown that individuals presenting with both ASD and psychosis may show less stereotypical sign of autism, such as repetitive or restrictive interest (Larson et al., 2017). The presentation of autism symptoms in this cohort may also make it less likely to be taken into account in treatment planning and case conceptualisation. New research has also found differences between those who present with early psychosis and those who present with early psychosis and co-occurring autism (ASD-P). Treise et al. (2021) found that people with autism and psychosis tended to exhibit a lot more social problems growing up, have a lot more persecutory delusions, and had their psychosis brought on by relationship difficulties than people who had psychosis but no autism. The differences in their presentation may also extend to their support needs. Helping to identify nuance in the presentation of clients in early psychosis services may also assist in case formulation and developing treatment plans for clients.

In acute and crisis mental health settings, there is also a tendency to respond swiftly with pharmacological interventions to contain the crisis and reduce distress. These first line medications, such as anti-psychotics and mood stabilisers (Shah et al., 2017) are often long-term treatments with a range of metabolic risk side-effects (Mazereel et al., 2020). Additionally, prescribing antipsychotic medication for individuals with ASD is increasingly seen as restrictive practice, particularly if behavioural interventions have not been tried first (Deb et al., 2024). For autism, there is no established medical ‘treatment’, and instead psychiatric medications, when prescribed, focus on alleviating symptoms of common secondary mental health challenges, such as depression, anxiety, and sleep difficulties.

In addition to medication differences, the focus for psychosocial interventions may also differ depending on whether looking through a psychiatric or neurodevelopmental lens. For bipolar disorder and psychosis, unusual behaviours (e.g. social withdrawal, compulsive and repetitive thoughts and behaviours, and unusual interpersonal style) may be pathologized and discouraged, and may be viewed as ‘early warning signs’ of a pending acute episode of illness. However, for autistic clients, the support paradigm shifts toward skill building to help the autistic person cope in a ‘neurotypical’ world, promoting flexibility in family and other systems (social, employment/training etc.) to understand and appreciate the unique qualities of the autistic person. Tailoring treatment plans for young people with signs of autism may be useful in developing the most effective treatment outcomes.

## Future Directions

Due to the potential risks and possible poorer prognosis of not considering autism and traits of autism in the formulation and treatment of young people suspected of having psychosis (Au-Yeung et al., 2019; Dossetor, 2007; Van Schalkwyk et al., 2015), it is important to consider implication for how services may change to address these potential risks. Two possible areas that may assist services in being more responsive to autism traits in their client presentations may be: (a) the use of language to include *traits of autism* and the capacity to discuss both *difference* and *distress* with families; and (b) the appropriate training and support for clinicians to identify and work with traits of autism in youth early psychosis settings.

### Developing Service Provision Language to Include Traits of Autism and Clinician Confidence to Discuss Both Difference and Distress with Families

While it remains important for psychiatry to identify clinically impairing ASD, it is important that clinicians can utilise a language to capture autism traits, including those which fall outside the current *impairment* definition required for definitive diagnosis. The absence of an established language to capture non-impairing autism creates a void which can become filled with less accurate diagnostic labels and the risk of unnecessary antipsychotic and mood-stabiliser medications.

A shared language to capture sub-clinical traits of autism could also improve clinical documentation and service delivery, as providing opportunities for clinicians to identify traits of autism in service level data collection may improve research and evaluation of these services. Terms such as ‘autism presentation without diagnosis’, ‘apparent autism’, ‘neuroatypicality’, and ‘autism phenotype without diagnosis’, if thoughtfully applied, could improve identification, support, and treatment in some cases.

Use of thoughtful language is not only important for clinical documentation, but also flows into the clinical dialogue with clients and families. When communicating with clients and families, mental health practitioners often prime the focus of the assessment toward ‘distress’ (problems, pain, impairment, weaknesses etc.). This does not encourage clients and families to identify ‘difference’ (not connecting with similar aged peers, experiencing the world differently to others) as a reason for seeking support. Normalising both *difference* and *distress* as appropriate reasons to seek support from a mental health service is essential to ensure clients and families are able to talk openly. More rounded assessments and improved client outcomes could be achieved through a clinician’s openness to explore both *difference* and *distress* as part of interventions. Importantly, even clinicians lacking skills and confidence to discuss and assess autism may feel more confident to introduce a conversation about ‘difference’.

### Training and Support for Clinicians to Identify Traits of Autism in Youth Early Psychosis Programs

Improving clinician skills and confidence in identifying autism presentations in youth mental health settings is important to ensure quality assessment and treatment. A focus on upskilling youth clinicians in client presentations which could be indicative of an underlying autism presentation may reduce the potential for symptoms of autism to be missed or overlooked. Psychosis services treating young people in the adolescent age range would benefit from including pathways for specialised primary and secondary consultations in situations in which traits of autism have been observed, even if there is no formal diagnosis. Skilled developmental assessments used in conjunction with more traditional cross-sectionally assessed information aligned with the current presentation, may help provide more efficacious formulation and treatment planning.

Training clinicians about autism traits and how these overlap with symptoms of psychotic illness and other mental health challenges could be an important step toward providing holistic and tailored supports and reduces the risks of unnecessarily medicalised treatment.

## Conclusion

As rates of autism and autism traits in the community continue to rise, there is a pressing need for mental health services to provide nuanced, tailored, and individualised care to autistic young people, young people with serious mental illness, and young people with both these conditions. Teasing out the different aetiologies can be complex and time-consuming and may be difficult to achieve for clinical staff who are usually trained in one approach or the other. However, providing a new language to talk about autism as well as more training to improve the understanding of autism and autism traits among mental health clinicians, may lead to improved assessment, formulation, diagnosis, and prognosis for these young people, which may be critical to improving outcomes for both the young person and their family, friends, and community.

## References

[CR1] Aggarwal, S., & Angus, B. (2015). Misdiagnosis versus missed diagnosis: Diagnosing autism spectrum disorder in adolescents. *Australasian Psychiatry,**23*(2), 120–123. 10.1177/1039856214568214 25653302 10.1177/1039856214568214

[CR2] American Psychiatric Association. (2013). *Diagnostic and statistical manual of mental disorders: DSM-5* (Vol. 5). American Psychiatric Association.

[CR3] Au-Yeung, S. K., Bradley, L., Robertson, A. E., Shaw, R., Baron-Cohen, S., & Cassidy, S. (2019). Experience of mental health diagnosis and perceived misdiagnosis in autistic, possibly autistic and non-autistic adults. *Autism,**23*(6), 1508–1518. 10.1177/1362361318818167 30547677 10.1177/1362361318818167

[CR4] Barlati, S., Minelli, A., Ceraso, A., Nibbio, G., Carvalho Silva, R., Deste, G., Turrina, C., & Vita, A. (2020). Social cognition in a research domain criteria perspective: A bridge between schizophrenia and autism spectra disorders. *Frontiers in Psychiatry,**11*, 806. 33005149 10.3389/fpsyt.2020.00806 PMC7485015

[CR5] Belcher, H. L., Morein-Zamir, S., Mandy, W., & Ford, R. M. (2022). Camouflaging intent, first impressions, and age of ASC diagnosis in autistic men and women. *Journal of Autism and Developmental Disorders,**52*(8), 3413–3426. 10.1007/s10803-021-05221-3 34342806 10.1007/s10803-021-05221-3 PMC9296412

[CR6] Ben-Sasson, A., Hen, L., Fluss, R., Cermak, S. A., Engel-Yeger, B., & Gal, E. (2009). A meta-analysis of sensory modulation symptoms in individuals with autism spectrum disorders. *Journal of Autism and Developmental Disorders,**39*(1), 1–11. 10.1007/s10803-008-0593-3 18512135 10.1007/s10803-008-0593-3

[CR7] Bottini, S. B., Morton, H. E., Buchanan, K. A., & Gould, K. (2024). Moving from disorder to difference: A systematic review of recent language use in autism research. *Autism Adulthood,**17*, 128–140. 10.1089/aut.2023.0030 10.1089/aut.2023.0030 PMC11319857 39144072

[CR8] Brede, J., Babb, C., Jones, C., Elliott, M., Zanker, C., Tchanturia, K., Serpell, L., Fox, J., & Mandy, W. (2020). “For me, the anorexia is just a symptom, and the cause is the autism”: Investigating restrictive eating disorders in autistic women. *Journal of Autism and Developmental Disorders,**50*, 4280–4296. 32274604 10.1007/s10803-020-04479-3 PMC7677288

[CR9] Bryson, S. E., Rogers, S. J., & Fombonne, E. (2003). Autism spectrum disorders: Early detection, intervention, education, and psychopharmacological management. *The Canadian Journal of Psychiatry,**48*(8), 506–516. 10.1177/070674370304800802 14574826 10.1177/070674370304800802

[CR10] Cappon, D. (1953). Clinical manifestations of autism and schizophrenia in childhood. *Canadian Medical Association Journal,**69*, 44–49. 13059732 PMC1822879

[CR11] Carpita, B., Muti, D., Cremone, I., Fagiolini, A., & Dell’Osso, L. (2022). Eating disorders and autism spectrum: Links and risks. *CNS Spectrums,**27*(3), 272–280. 10.1017/S1092852920002011 33161925 10.1017/S1092852920002011

[CR12] Centre of Disease Control and Prevention. (2024). *Autism spectrum disorder (ASD)—Data & statistics on autism spectrum disorder*. https://www.cdc.gov/ncbddd/Autism/data.html

[CR13] Chisholm, K., Pelton, M., Duncan, N., Kidd, K., Wardenaar, K. J., Upthegrove, R., Broome, M. R., Lin, A., & Wood, S. J. (2019). A cross-sectional examination of the clinical significance of autistic traits in individuals experiencing a first episode of psychosis. *Psychiatry Research,**282*, 112623. 10.1016/j.psychres.2019.112623 31685288 10.1016/j.psychres.2019.112623

[CR14] Colizzi, M., Bortoletto, R., Costa, R., Bhattacharyya, S., & Balestrieri, M. (2022). The autism–psychosis continuum conundrum: Exploring the role of the endocannabinoid system. *International Journal of Environmental Research and Public Health,**19*(9), 5616. 10.3390/ijerph19095616 35565034 10.3390/ijerph19095616 PMC9105053

[CR15] Corden, K., Brewer, R., & Cage, E. (2022). A systematic review of healthcare professionals’ knowledge, self-efficacy and attitudes towards working with autistic people. *Review Journal of Autism and Developmental Disorders,**9*, 386–399. 10.1007/s40489-021-00263-w

[CR16] De Berardis, D., De Filippis, S., Masi, G., Vicari, S., & Zuddas, A. (2021). A neurodevelopment approach for a transitional model of early onset schizophrenia. *Brain Sciences,**11*(2), 275. 10.3390/brainsci11020275 33672396 10.3390/brainsci11020275 PMC7926620

[CR17] Deb, S., Limbu, B., Bianco, A., & Bertelli, M. (2024). Ethical prescribing of psychotropic medications for people with neurodevelopmental disorders. *Advances in Neurodevelopmental Disorders,**8*(1), 198–207. 10.1007/s41252-023-00365-y

[CR18] Defresne, P., & Mottron, L. (2022). Clinical situations in which the diagnosis of autism is debateable: An analysis and recommendations. *Canadian Journal of Psychiatry,**67*(5), 331–335. 10.1177/07067437211041469 34482753 10.1177/07067437211041469 PMC9065488

[CR19] Dell’Osso, L., Lorenzi, P., & Carpita, B. (2019). Autistic traits and illness trajectories. *Clinical Practice and Epidemiology in Mental Health,**15*, 94–98. 10.2174/1745017901915010094 31819756 10.2174/1745017901915010094 PMC6882132

[CR20] Dossetor, D. R. (2007). ‘All that glitters is not gold’: Misdiagnosis of psychosis in pervasive developmental disorders—A case series. *Clinical Child Psychology and Psychiatry,**12*(4), 537–548. 10.1177/1359104507078476 18095536 10.1177/1359104507078476

[CR21] Elder, J. H., Kreider, C. M., Brasher, S. N., & Ansell, M. (2017). Clinical impact of early diagnosis of autism on the prognosis and parent-child relationships. *Psychology Research and Behavior Management,**10*, 283–292. 10.2147/PRBM.S117499 28883746 10.2147/PRBM.S117499 PMC5576710

[CR22] Ferrara, M., Curtarello, E. M. A., Simonelli, G., Yoviene Sykes, L. A., Fusar-Poli, L., Folesani, F., & Grassi, L. (2024). First-episode psychosis and autism spectrum disorder: A scoping review and a guide to overcome diagnostic challenges. *International Journal of Mental Health,**53*(1), 4–35. 10.1080/00207411.2023.2276872

[CR23] Gallant, C., Roudbarani, F., Ibrahim, A., Maddox, B. B., & Weiss, J. A. (2023). Clinician knowledge, confidence, and treatment practices in their provision of psychotherapy to autistic youth and youth with ADHD. *Journal of Autism and Developmental Disorders,**53*, 4214–4228. 10.1007/s10803-022-05722-9 36076117 10.1007/s10803-022-05722-9 PMC10539421

[CR24] Gotham, K., Bishop, S. L., Hus, V., Huerta, M., Lund, S., Buja, A., & Lord, C. (2013). Exploring the relationship between anxiety and insistence on sameness in autism spectrum disorders. *Autism Research,**6*(1), 33–41. 10.1002/aur.1263 23258569 10.1002/aur.1263 PMC4373663

[CR25] Guerra, S., Pontillo, M., Chieppa, F., Passarini, S., Di Vincenzo, C., Casula, L., Di Luzio, M., Valeri, G., & Vicari, S. (2024). Autism spectrum disorder and early psychosis: A narrative review from a neurodevelopmental perspective. *Frontiers in Psychiatry*. 10.3389/fpsyt.2024.1362511 38571993 10.3389/fpsyt.2024.1362511 PMC10987738

[CR26] Gupta, S., & Kulhar, P. (2010). What is schizophrenia: A neurodevelopmental or neurodegenerative disorder or a combination of both? A critical analysis. *Indian Journal of Psychiatry,**52*(1), 21–27. 10.4103/0019-5545.58891 20174514 10.4103/0019-5545.58891 PMC2824976

[CR27] Healey, K. M., Bartholomeusz, C., & Penn, D. L. (2016). Deficits in social cognition in first episode psychosis: A review of the literature. *Clinical Psychology Review,**50*, 108–137. 10.1016/j.cpr.2016.10.001 27771557 10.1016/j.cpr.2016.10.001

[CR28] Howes, A. E., Burns, M. E., & Surtees, A. D. (2021). Barriers, facilitators, and experiences of the autism assessment process: A systematic review of qualitative research with health professionals. *Professional Psychology: Research and Practice,**52*(5), 449.

[CR29] Huang, Y., Arnold, S. R. C., Foley, K. R., & Trollor, J. N. (2022). Choose your own adventure: Pathways to adulthood autism diagnosis in Australia. *Journal of Autism and Developmental Disorders,**52*(2984), 2996. 10.1007/s10803-021-05169-4 10.1007/s10803-021-05169-4 34241747

[CR30] Hus, Y., & Segal, O. (2021). Challenges surrounding the diagnosis of autism in children. *Neuropsychiatric Disease and Treatment,**3*(7), 3509–3529. 10.2147/NDT.S282569 10.2147/NDT.S282569 PMC8654688 34898983

[CR31] Iversen, S., & Kildahl, A. N. (2022). Case report: Mechanisms in misdiagnosis of autism as borderline personality disorder. *Frontiers in Psychology,**13*, 735205. 10.3389/fpsyg.2022.735205 35185714 10.3389/fpsyg.2022.735205 PMC8855062

[CR32] Jack, J. (2014). *Autism and gender: From refrigerator mothers to computer geeks*. University of Illinois Press.

[CR33] James, S. N., & Smith, C. J. (2020). Early autism diagnosis in the primary care setting. *Seminars in Pediatric Neurology,**35*, 100827. 10.1016/j.spen.2020.100827 32892954 10.1016/j.spen.2020.100827

[CR34] Jellett, R., & Muggleton, J. (2022). Implications of applying “clinically significant impairment” to autism assessment: Commentary on six problems encountered in clinical practice. *Journal of Autism and Developmental Disorders,**52*(3), 1412–1421. 10.1007/s10803-021-04988-9 33893595 10.1007/s10803-021-04988-9

[CR35] Kincaid, D. L., Doris, M., Shannon, C., & Mulholland, C. (2017). What is the prevalence of autism spectrum disorder and ASD traits in psychosis? A systematic review. *Psychiatry Research,**250*, 99–105. 10.1016/j.psychres.2017.01.017 28152400 10.1016/j.psychres.2017.01.017

[CR36] King, B. H., & Lord, C. (2011). Is schizophrenia on the autism spectrum? *Brain Research,**1380*, 34–41. 10.1016/j.brainres.2010.11.031 21078305 10.1016/j.brainres.2010.11.031

[CR37] Lai, M. C., & Baron-Cohen, S. (2015). Identifying the lost generation of adults with autism spectrum conditions. *The Lancet Psychiatry,**2*(11), 1013–1027. 10.1016/S2215-0366(15)00277-1 26544750 10.1016/S2215-0366(15)00277-1

[CR38] Landa, R. J., Gross, A. L., Stuart, E. A., & Faherty, A. (2013). Developmental trajectories in children with and without autism spectrum disorders: The first 3 years. *Child Development,**84*(2), 429–442. 23110514 10.1111/j.1467-8624.2012.01870.x PMC4105265

[CR39] Landry, O., & Chouinard, P. A. (2016). Why we should study the broader autism phenotype in typically developing populations. *Journal of Cognition and Development,**17*(4), 584–595. 10.1080/15248372.2016.1200046

[CR40] Larson, F. V., Wagner, A. P., Jones, P. B., Tantam, D., Lai, M. C., Baron-Cohen, S., & Holland, A. J. (2017). Psychosis in autism: Comparison of the features of both conditions in a dually affected cohort. *British Journal of Psychiatry,**210*(4), 269–275. 10.1192/bjp.bp.116.187682 10.1192/bjp.bp.116.187682 PMC5376719 27979819

[CR41] Lee, M., Nayar, K., Maltman, N., Hamburger, D., Martin, G. E., Gordon, P. C., & Losh, M. (2020). Understanding social communication differences in autism spectrum disorder and first-degree relatives: A study of looking and speaking. *Journal of Autism and Developmental Disorders,**50*, 2128–2141. 10.1007/s10803-019-03969-3 30864059 10.1007/s10803-019-03969-3 PMC7261276

[CR42] Lee, W. W. S. J., & Lee, S. Y. L. (2023). Diagnostic challenges in distinguishing autism spectrum disorder from psychosis: A case report. *European Psychiatry,**66*(S1), S1092–S1093. 10.1192/j.eurpsy.2023.2321

[CR43] Lipinski, S., Boegl, K., Blanke, E., Suenkel, U., & Dziobek, I. (2022). A blind spot in mental healthcare? Psychotherapists lack education and expertise for the support of adults on the autism spectrum. *Autism,**26*(6), 1509–1521. 10.1177/13623613211057973 34825580 10.1177/13623613211057973 PMC9344568

[CR44] Liu, S., Lin, K., Zhang, Y., Gao, Y., Wang, W., Du, M., Jiang, T., Zhou, M., & Zhang, X. (2023). Prevalence and risk factors of psychotic symptoms in middle-aged patients with first-episode drug-naïve major depressive disorder: A large-scale cross-sectional study. *Journal of Affective Disorders*, *325*, 102–109. 10.1016/j.jad.2023.01.002 10.1016/j.jad.2023.01.002 36623569

[CR45] Luciano, C. C., Keller, R., Politi, P., Aguglia, E., Magnano, F., Burti, L., Muraro, F., Aresi, A., Damiani, S., & Berardi, D. (2014). Misdiagnosis of high function autism spectrum disorders in adults: An Italian case series. *Autism,**4*, 131. 10.4172/2165-7890.1000131

[CR46] Maddox, B. B., Crabbe, S., Beidas, R. S., Brookman-Frazee, L., Cannuscio, C. C., Miller, J. S., Nicolaidis, C., & Mandell, D. S. (2020). “I wouldn’t know where to start”: Perspectives from clinicians, agency leaders, and autistic adults on improving community mental health services for autistic adults. *Autism,**24*(4), 919–930. 10.1177/1362361319882227 31674198 10.1177/1362361319882227 PMC7192780

[CR47] Martinez, M., Thomas, K. C., Williams, C. S., Christian, R., Crais, E., Pretzel, R., & Hooper, S. R. (2018). Family experiences with the diagnosis of autism spectrum disorder: System barriers and facilitators of efficient diagnosis. *Journal of Autism and Developmental Disorders,**48*, 2368–2378. 10.1007/s10803-018-3493-1 29453706 10.1007/s10803-018-3493-1

[CR48] Mazereel, V., Detraux, J., Vancampfort, D., van Winkel, R., & De Hert, M. (2020). Impact of psychotropic medication effects on obesity and the metabolic syndrome in people with serious mental illness. *Frontiers in Endocrinology (Lausanne)*. 10.3389/fendo.2020.573479 10.3389/fendo.2020.573479 PMC7581736 33162935

[CR49] Morimoto, Y., Yamamoto, N., Kanegae, S., Matsuzaka, R., & Imamura, A. (2021). Genetic overlap among autism spectrum disorders and other neuropsychiatric disorders. In A. M. Grabrucker (Ed.), *Autism spectrum disorders. *Exon Publications. 10.36255/exonpublications 34495616

[CR50] Mottron, L., & Bzdok, D. (2020). Autism spectrum heterogeneity: Fact or artifact? *Molecular Psychiatry,**25*, 3178–3185. 10.1038/s41380-020-0748-y 32355335 10.1038/s41380-020-0748-y PMC7714694

[CR51] Park, J. S., Damme, K. S. F., Kuhney, F. S., & Mittal, V. A. (2022). Anxiety symptoms, rule learning, and cognitive flexibility in non-clinical psychosis. *Science and Reports,**12*, 5649. 10.1038/s41598-022-09620-z 10.1038/s41598-022-09620-z PMC8983653 35383232

[CR52] Picci, G., & Scherf, K. S. (2015). A two-hit model of Autism: Adolescence as the second hit. *Clinical Psychological Science,**3*(3), 349–371. 10.1177/2167702614540646 26609500 10.1177/2167702614540646 PMC4655890

[CR53] Ribolsi, M., Fiori Nastro, F., Pelle, M., Medici, C., Sacchetto, S., Lisi, G., Riccioni, A., Siracusano, M., Mazzone, L., & Di Lorenzo, G. (2022). Recognizing psychosis in autism spectrum disorder. *Frontiers in Psychiatry,**13*, 768586. 10.3389/fpsyt.2022.768586 35295770 10.3389/fpsyt.2022.768586 PMC8918655

[CR54] Rim, S., Kwak, K., & Park, S. (2023). Risk of psychiatric comorbidity with autism spectrum disorder and its association with diagnosis timing using a nationally representative cohort. *Research in Autism Spectrum Disorders*. 10.1016/j.rasd.2023.102134

[CR55] Rosen, T. E., Mazefsky, C. A., Vasa, R. A., & Lerner, M. D. (2018). Co-occurring psychiatric conditions in autism spectrum disorder. *International Review of Psychiatry,**30*(1), 40–61. 10.1080/09540261.2018.1450229 29683351 10.1080/09540261.2018.1450229

[CR56] Schäfer, I., & Fisher, H. L. (2011). Childhood trauma and psychosis—What is the evidence? *Dialogues in Clinical Neuroscience,**13*(3), 360–5. 10.31887/DCNS.2011.13.2/ischaefer 22033827 10.31887/DCNS.2011.13.2/ischaefer PMC3182006

[CR57] Schroeder, K., Fisher, H. L., & Schäfer, I. (2013). Psychotic symptoms in patients with borderline personality disorder: Prevalence and clinical management. *Current Opinion in Psychiatry,**26*(1), 113–119. 23168909 10.1097/YCO.0b013e32835a2ae7

[CR58] Seikkula, J. (2019). Psychosis is not illness but a survival strategy in severe stress: A proposal for an addition to a phenomenological point of view. *Psychopathology,**52*(2), 143–150. 10.1159/000500162 31362287 10.1159/000500162

[CR59] Shah, N., Sandeep Grover, S., & Prasad Rao, G. (2017). Clinical practice guidelines for management of bipolar disorder. *Indian Journal of Psychiatry,**59*(1), S51–S66. 10.4103/0019-5545.196974 28216785 10.4103/0019-5545.196974 PMC5310104

[CR60] Sinclair, D., Oranje, B., Razak, K. A., Siegel, S. J., & Schmid, S. (2017). Sensory processing in autism spectrum disorders and Fragile X syndrome—From the clinic to animal models. *Neuroscience and Biobehavioral Reviews,**76*(Pt B), 235–253. 10.1016/j.neubiorev.2016.05.029 27235081 10.1016/j.neubiorev.2016.05.029 PMC5465967

[CR61] Spauwen, J., Krabbendam, L., Lieb, R., Wittchen, H. U., & Van Os, J. (2006). Impact of psychological trauma on the development of psychotic symptoms: Relationship with psychosis proneness. *The British Journal of Psychiatry,**188*(6), 527–533. 10.1192/bjp.bp.105.011346 16738342 10.1192/bjp.bp.105.011346

[CR62] Sunwoo, M., O’Connell, J., Brown, E., Lin, A., Wood, S. J., McGorry, P., & O’Donoghue, B. (2020). Prevalence and outcomes of young people with concurrent autism spectrum disorder and first episode of psychosis. *Schizophrenia Research,**216*, 310–315. 10.1016/j.schres.2019.11.037 31787480 10.1016/j.schres.2019.11.037

[CR63] Treise, C., Simmons, C., Marshall, N., Painter, M., & Perez, J. (2021). Autism spectrum disorder in early intervention in psychosis services: Implementation and findings of a 3-step screening and diagnostic protocol. *Journal of Psychiatric Practice,**27*(1), 23–32. 10.1097/PRA.0000000000000525 33438864 10.1097/PRA.0000000000000525

[CR64] Van Schalkwyk, G. I., Peluso, F., Qayyum, Z., McPartland, J. C., & Volkmar, F. R. (2015). Varieties of misdiagnosis in ASD: An illustrative case series. *Journal of Autism and Developmental Disorders,**45*(4), 911–918. 10.1007/s10803-014-2239-y 25218849 10.1007/s10803-014-2239-y

[CR65] Van Steensel, F. J., Bögels, S. M., & Perrin, S. (2011). Anxiety disorders in children and adolescents with autistic spectrum disorders: A meta-analysis. *Clinical Child and Family Psychology Review,**14*(3), 302–317. 10.1007/s10803-008-0593-3 21735077 10.1007/s10567-011-0097-0 PMC3162631

[CR66] Verdoux, H., & van Os, J. (2002). Psychotic symptoms in non-clinical populations and the continuum of psychosis. *Schizophrenia Research,**54*(1–2), 59–65. 10.1016/S0920-9964(01)00352-8 11853979 10.1016/s0920-9964(01)00352-8

[CR67] Volkmar, F. R. (2016). From infantile autism to autism spectrum. Evolution of the diagnostic concept. In C. McDougle (Ed.), *Autism spectrum disorder* (pp. 3–18). Oxford University Press.

[CR68] Volkmar, F. R., & McPartland, J. C. (2014). From Kanner to DSM-5: Autism as an evolving diagnostic concept. *Annual Review of Clinical Psychology,**10*, 193–212. 10.1146/annurev-clinpsy-032813-153710 24329180 10.1146/annurev-clinpsy-032813-153710

[CR69] West, M. L., Guest, R. M., & Carmel, A. (2021). Comorbid early psychosis and borderline personality disorder: Conceptualizing clinical overlap, etiology, and treatment. *Personal Mental Health,**15*(3), 208–222. 10.1002/pmh.1509 10.1002/pmh.1509 33955194

[CR70] Winters, N. C., Hanson, G., & Stoyanova, V. (2007). The case formulation in child and adolescent psychiatry. *Child and Adolescent Psychiatric Clinics of North America,**16*(1), 111–32. 10.1016/j.chc.2006.07.010 17141121 10.1016/j.chc.2006.07.010

[CR71] Yirmiya, N., Gamliel, I., Pilowsky, T., Feldman, R., Baron-Cohen, S., & Sigman, M. (2006). The development of siblings of children with autism at 4 and 14 months: Social engagement, communication, and cognition. *Journal of Child Psychology and Psychiatry,**47*(5), 511–523. 10.1111/j.1469-7610.2005.01528.x 16671934 10.1111/j.1469-7610.2005.01528.x

[CR72] Zheng, Z., Zheng, P., & Zou, X. (2018). Association between schizophrenia and autism spectrum disorder: A systematic review and meta-analysis. *Autism Research,**11*(8), 1110–1119. 10.1002/aur.1977 30284394 10.1002/aur.1977

